# Predicting CaO activity in multiple slag system using improved whale optimization algorithm and categorical boosting

**DOI:** 10.1038/s41598-025-93980-9

**Published:** 2025-03-19

**Authors:** Zi-cheng Xin, Jiang-shan Zhang, Qing Liu

**Affiliations:** 1 State Key Laboratory of Advanced Metallurgy, University of Science and Technology Beijing, Beijing, 100083 China; 2 School of Automation and Electrical Engineering, University of Science and Technology Beijing, Beijing, 100083 China

**Keywords:** Multiple slag system, *a*(CaO), FactSage, Ion and molecule coexistence theory, Improved whale optimization algorithm, Categorical boosting, Engineering, Mathematics and computing

## Abstract

The activity of slag components is one of the primary factors influencing the thermodynamic properties of slag. In this study, a feasible model was established to predict the *a*(CaO) using improved whale optimization algorithm (IWOA) and Categorical Boosting (CatBoost). The effects of other variables on *a*(CaO) were listed in descending order of influence as follows: *w*(CaO), *w*(SiO_2_), temperature, *w*(MgO), and *w*(Al_2_O_3_). And the IWOA-CatBoost model achieved the highest R^2^ value of 0.9200, lowest RMSE of 0.0042, and lowest MAE of 0.0030 in predicting the *a*(CaO). The performance of the optimal IWOA-CatBoost model was evaluated and compared with that of known models. The results demonstrate that the IWOA-CatBoost model outperformed existing models and methods, such as the Factsage, ion and molecule coexistence theory, and genetic algorithm—backpropagation neural network. The accurate calculation of slag component activity is of great significance to the analysis of the thermodynamic properties of slag. Meanwhile, the approach and algorithm used to develop the a(CaO) prediction model can also be applied to predicting the activity of other slag components or other metallurgical applications (e.g., predicting molten steel temperature, steel composition, and alloy yield).

## Introduction

With the advancement of the steel industry toward higher-end, smarter and greener production, the requirements for product quality continue to increase^1^. Slag has a significant impact on the quality of molten steel, and desulfurization is one of its primary tasks in LF refining process^2–4^. The desulfurization reaction primarily occurs through interfacial reactions between the steel and slag, and the activity of each slag component significantly impacts the desulfurization process. In addition, activity theory can explain a range of critical phenomena in metallurgical processes, such as phase equilibria and phase transformations, element migration, and the direction of chemical reactions. With the deepening research on physicochemical properties of slag, the research on slag component activity has been paid more attention by metallurgists.

Metallurgists have developed a range of thermodynamic models for molten slag, including the complete ionic solution model^5^, the regular solution model^6^, the new generation solution geometrical model^7^, and the ion and molecule coexistence theory (IMCT)^8^. Chang et al.^9^ calculated the liquidus temperature, activity, and cooling crystallization process of slag using the FactSage thermodynamic software, and analyzed the effect mechanism of Al_2_O_3_ on the slag viscosity. Tang et al.^10^ calculated the slag components activity in the CaO–MgO–Al_2_O_3_–SiO_2_ refining slag system to explore the thermodynamic equilibrium relationships among refining slag, molten steel, and inclusions using the FactSage. Guo et al.^11^ developed an activity calculation model for the CaO–SiO_2_–MgO–Al_2_O_3_ slag system based on the IMCT, and validated this model using experimental data. However, FactSage thermodynamic software typically assumes that reactions reach equilibrium. In actual metallurgical processes, the reactions between slag and metal do not always achieve thermodynamic equilibrium, especially under conditions of rapid reactions or non-equilibrium states. As a result, the calculated activity value may not accurately reflect situation in actual process. Additionally, most models and methods involve assumed conditions and overlook the effects of interactions between components on activity, resulting in deviations between calculated and experimental values. Therefore, the thermodynamic model should be further developed and improved^11^.

With the rapid development of machine learning theory, Wu et al.^12^ established an activity prediction model for multiple slag systems based on a genetic algorithm (GA)—back propagation (BP) neural network algorithm and demonstrated a good agreement between this model’s calculated values and experimental values. However, the study validated the accuracy of the constructed model solely by comparing calculated values with literature values in graphical form, without conducting a comprehensive comparative analysis with known models and methods, and employed the traditional GA-BP neural network algorithm. Since the emergence of deep learning, deep neural network has been widely applied across diverse industries^13^. However, nearly all deep neural network algorithms currently require large datasets for effective training. In fact, challenges such as limited data availability and high data collection costs are common, so small sample datasets learning is particularly important. Categorical Boosting (CatBoost), an improved decision tree algorithm, was applied to the prediction of *a*(CaO) in multiple slag system with small sample datasets in this study. The CatBoost algorithm can achieve high predictive accuracy with small sample set^14^. Jin and Gu et al.^15,16^ validated the feasibility of CatBoost for small-sample prediction in the contexts of the blasting fragment large block percentage ratio (a regression study with 36 data samples) and fault diagnosis of photovoltaic array (a classification study with 55 training samples and 110 training samples), respectively.

Based on the aforementioned analysis, the data of *a*(CaO) obtained using the same activity measurement method was first collected in this study. Next, a correlation analysis was performed to assess the effect of various factors on *a*(CaO). Furthermore, the convergence factor of the standard whale optimization algorithm was improved to enhance the global search ability in the early stage and the local optimization speed in the later stage. Then, a prediction model of *a*(CaO) was established based on the improved whale optimization algorithm (IWOA)—CatBoost. Finally, various statistical evaluation metrics were employed to compare and assess the established model against existing models and methods (such as FactSage, IMCT, GA-BP), demonstrating the accuracy of the established model. Meanwhile, the modeling approach presented in this study can also be applied to predicting the activity of other slag components.

## Data collection and data analysis

Slag is a multi-component melt mainly composed of different oxides and is a typical by-product in steelmaking. Refining slag plays an important role in the steelmaking process, as sulfur is primarily removed from the molten steel through interfacial chemical reaction of the steel-slag. Meanwhile, the activity of CaO in the slag also has a significant impact on the content of CaO in inclusions^17,18^. Therefore, studying the activity of slag components is of great significance. The following analysis, based on metallurgical mechanisms, examines the impact of various factors (temperature and slag composition, including CaO, SiO_2_, MgO, and Al_2_O_3_ content) on CaO activity, desulfurization reactions, and inclusion control.

In terms of the impact of different factors on CaO activity, as the temperature increases, the activity of CaO increases. The possible reason is that SiO_2_, as a reactant, always involves CaO in the reaction. According to Le Chatelier’s principle and IMCT, compounds such as CaSiO_3_ and Ca_2_SiO_4_ decompose to form Ca^2+^ and O^2−^, which leads to an increase in the activity of Ca^2+^ and O^2−^, thus raising the activity of CaO^19^. As the CaO content increases, the activity of CaO also increases. Meanwhile, a large amount of CaO exists in the slag in the form of Ca^2+^ and O^2−^, which causes the activity of other components in the slag to gradually decrease^17^. As the SiO_2_ content increases, the activity of CaO gradually decreases^19^. Under constant basicity, the activity of CaO first increases and then decreases as the MgO content increases^20^. As the Al_2_O_3_ content increases, the activity of CaO decreases. Under basic slag conditions, Al_2_O_3_ is acidic. The combination of Al_2_O_3_ and CaO forms the CaO·Al_2_O_3_ compound, which reduces the free CaO content in the slag. Meanwhile, some of the free O^2-^ is consumed when forming aluminates, leading to a decrease in the CaO activity in the slag^21^.

In terms of the influence of various factors on desulfurization reactions and inclusion control, as the CaO content increases, more O^2-^ is provided, the optical basicity increases, the sulfur capacity of the slag increases, and the sulfur distribution ratio between steel and slag increases, all of which facilitate desulfurization^22^. Under a fixed Al_2_O_3_ content, as the CaO content increases, the CaO activity increases while the Al_2_O_3_ activity decreases, which facilitates the slag’s adsorption of Al_2_O_3_ inclusions and enhances its deoxidation capacity. However, if the CaO content becomes excessively high, the increased CaO activity results in a higher CaO content in the inclusions^23^. Meanwhile, an excessively high CaO content leads to the precipitation of solid phase particles from the slag, which increases the viscosity, reduces fluidity, and deteriorates the desulfurization kinetics of slag^24^. Under basic slag conditions, SiO_2_ is a stronger acidic oxide than Al_2_O_3_, and an excess of SiO_2_ results in a decline in the desulfurization efficiency of the slag. Under specific conditions, SiO_2_ increases the viscosity of the slag and decreases its surface tension. To promote the infiltration, adsorption, and dissolution of inclusions, it is crucial to minimize the surface tension of the slag while ensuring that the viscosity remains stable^19^. MgO, being a basic oxide, can provide O^2-^, but its desulfurization capacity is slightly lower than that of CaO^24^. Under certain conditions, as the MgO content increases, the desulfurization capacity of the slag improves. However, when the MgO content surpasses a certain threshold, further increases lead to a decrease in desulfurization capacity. This is due to the high melting point of MgO (2800 °C), which, with increasing content, results in reduced slag fluidity and deteriorated desulfurization kinetics. Al_2_O_3_ itself lacks desulfurization capacity. In basic slags, Al_2_O_3_ acts as an acidic oxide. As the Al_2_O_3_ content increases, the effective CaO content in the slag decreases, leading to a reduced desulfurization capacity. This also hampers the removal of Al_2_O_3_ inclusions from the molten steel^22^. Increasing the Al_2_O_3_ content within a certain range can reduce the viscosity of the slag, improve its fluidity, and enhance the desulfurization kinetics^25^.

For the determination of slag component activity, commonly used experimental methods include the vapor pressure method, chemical equilibrium method, partition coefficient method, and electromotive force method. Metallurgical researchers usually use the chemical equilibrium method to measure slag component activity. Accordingly, 123 experimental data sets of *a*(CaO) measured by this method were collected for modeling research, as shown in Table 1

**Table 1:** Experimental data used for the calculation of *a*(CaO).

Slag system	Number of data	References
CaO–SiO_2_	12	^26^
CaO–SiO_2_–Al_2_O_3_	47	^26^
CaO–SiO_2_–MgO	25	^26^
CaO–SiO_2_–MgO–Al_2_O_3_	12 + 15 + 12	^21,26,27^

**Fig. 1:**
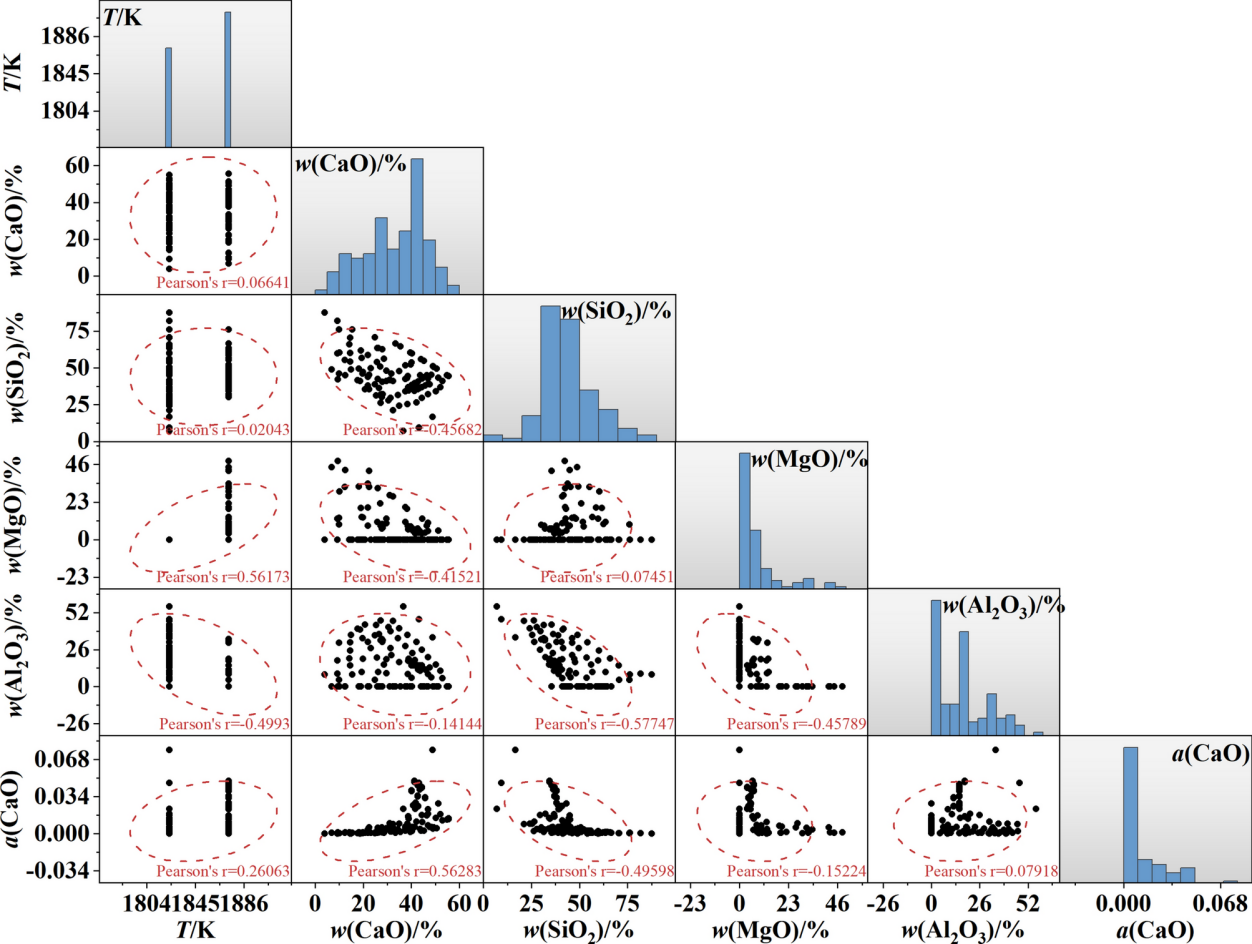
Scatter matrix diagram of temperature (*T*), *w*(CaO), *w*(SiO_2_), *w*(MgO), and *w*(Al_2_O_3_). Note: *w* represents the weight percent of slag components; *a*(CaO) denotes the activity of CaO.

A scatter matrix diagram and a Pearson correlation coefficient were used to visualize the data sets and reflect the correlation between these data, as shown in Fig. 1. In Fig. 1, the histograms along the diagonal display the distribution of each individual variable, while the scatter plots in the lower triangles illustrate the relationships between pairs of variables. For example, the left-most plot in the bottom row of Fig. 1 shows the relationship between temperature and the *a*(CaO). The temperature values were 1823 K and 1873 K. The range of the *w*(CaO), *w*(SiO_2_), *w*(MgO), and *w*(Al_2_O_3_) was 3.9–55.6%, 7.1–87.6%, 0–48.2%, and 0–56.2%, respectively. Meanwhile, the effects of other variables on *a*(CaO) are listed in descending order of influence as follows: *w*(CaO), *w*(SiO_2_), temperature, *w*(MgO), and *w*(Al_2_O_3_). The *a*(CaO) increased with the increase of the temperature. A higher *w*(CaO) was beneficial to improve the *a*(CaO). The *w*(SiO_2_) has a negative effect on the *a*(CaO). Compared to *w*(CaO), *w*(SiO_2_), and temperature, *w*(MgO) and *w*(Al_2_O_3_) have a smaller impact on *a*(CaO).

## Establishment of a(CaO) prediction model

**Fig. 2:**
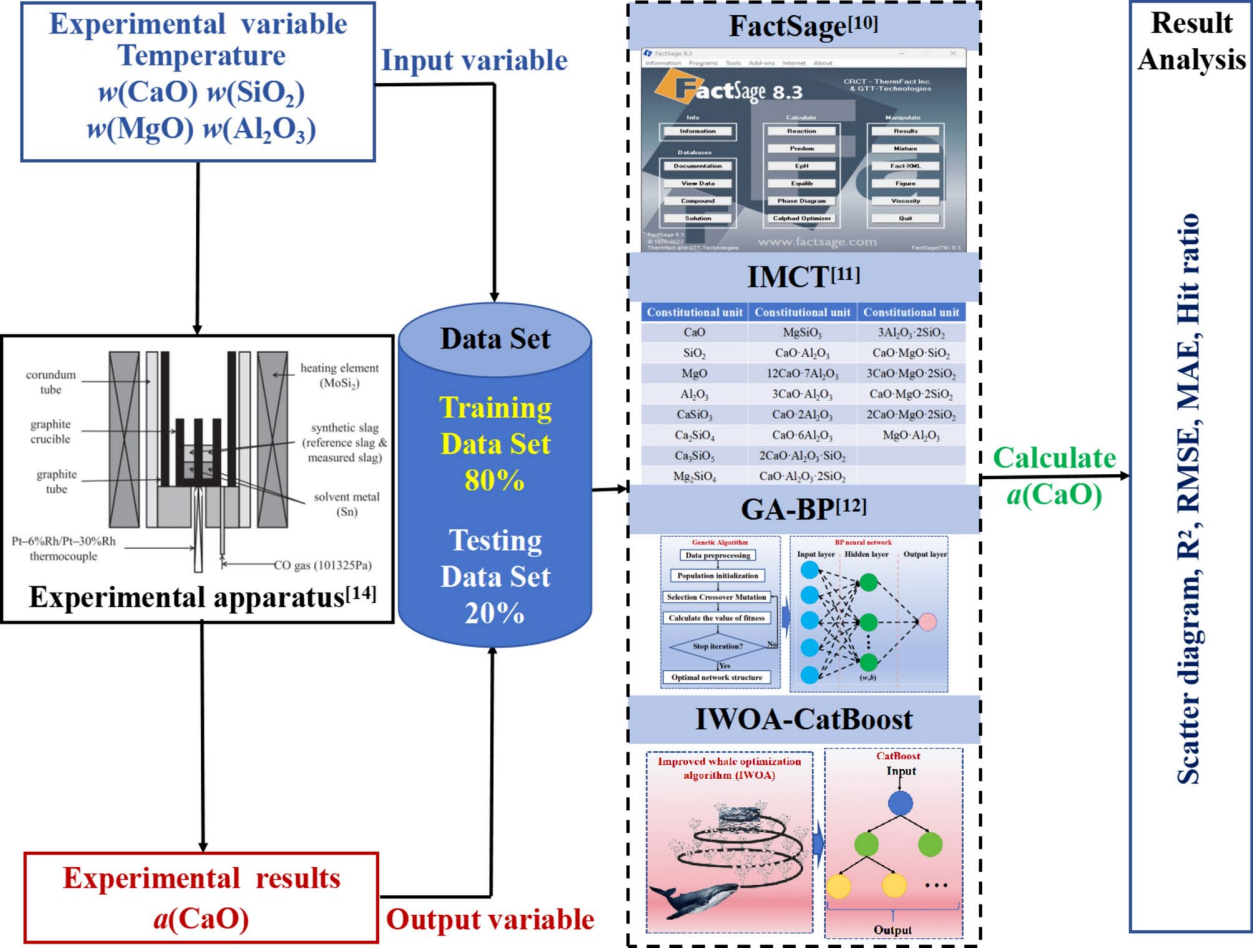
Modeling workflow.

In this study, the data set was first randomly divided into a training data set (80%) and a testing data set (20%). Then, an *a*(CaO) calculation model based on IWOA-CatBoost was developed using the training data set. Subsequently, based on the same testing data set, the calculated values of *a*(CaO) were obtained using FactSage^10^, IMCT^11^, GA-BP neural network algorithm^12^, and IWOA-CatBoost. Finally, the accuracy of the established model was evaluated using R^2^, RMSE, MAE, and scatter plots. The modeling workflow is shown in Fig. 2.

**Fig. 3:**
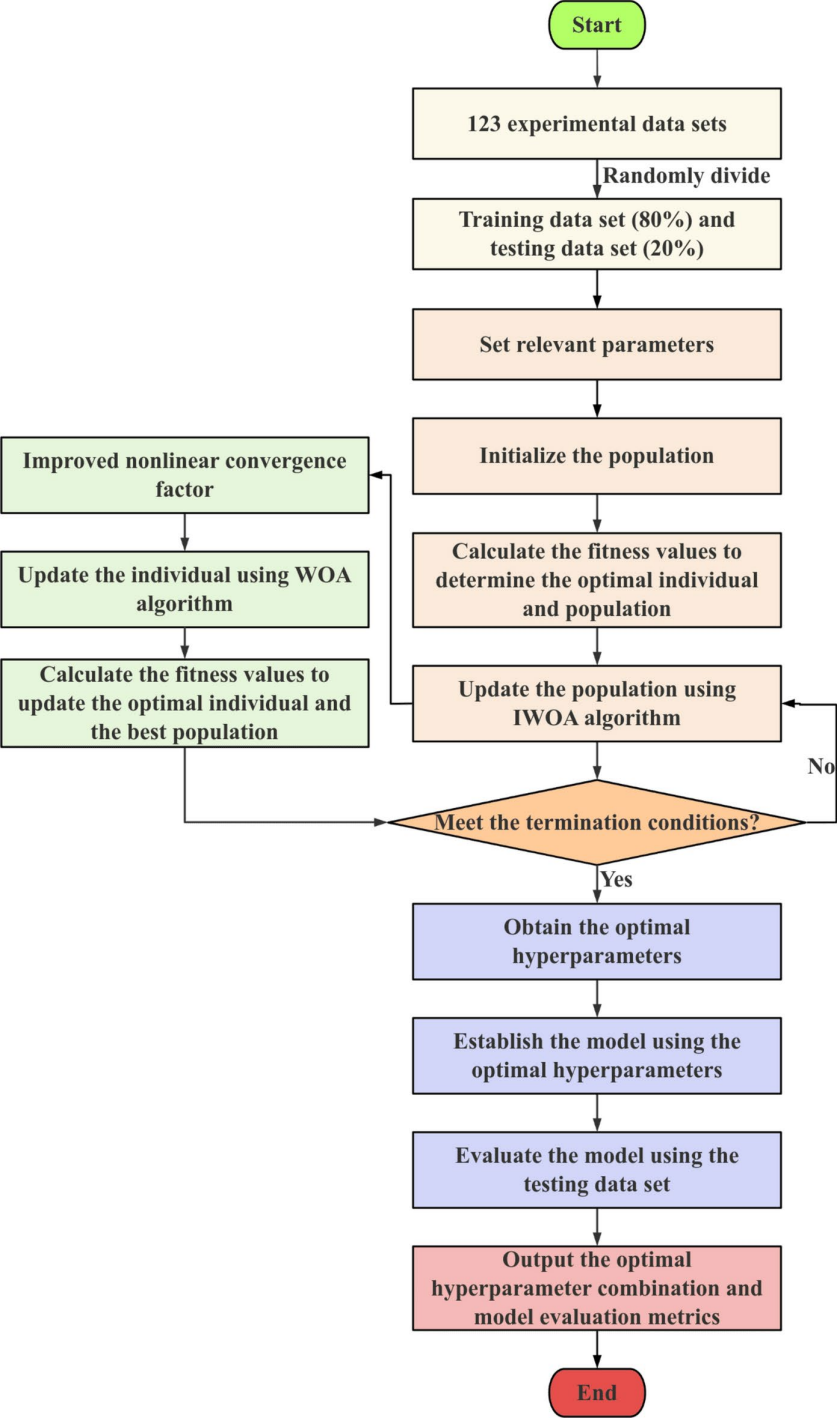
IWOA-CatBoost modeling flowchart.

Figure 3 presents the IWOA-CatBoost modeling flowchart. The specific steps of the IWOA-CatBoost modeling are outlined as follows:

1. Collect CaO activity data under different factors from existing literature (a total of 123 data sets);
2. Divide the 123 experimental data sets into a training data set (80%) and a testing data set (20%), with factors such as temperature, *w*(CaO), *w*(SiO_2_), *w*(MgO), and *w*(Al_2_O_3_) as input variables, and CaO activity as the output variable of the model;
3. Set the whale population size *n*, maximum number of iterations *max_iter*, and define the value ranges for the CatBoost hyperparameters: learning_rate, depth, n_estimators, l2_leaf_reg, subsample, bagging_temperature, and colsample_bylevel;
4. Set the CatBoost hyperparameters to each whale individual and initialize the whale population;
5. Calculate the fitness value of each whale individual to determine the current best individual and the optimal value of the whale population;
6. The improved IWOA algorithm is used to update the positions of the population individuals, updating parameters *a*, *D*, *A*, and *C*, where *a* represents the improved nonlinear convergence factor, which coordinates the algorithm’s global search and local optimization;
7. Calculate the fitness values, and through the comparison of fitness values, update the optimal solution for each whale individual and the optimal solution for the whale population, thereby obtaining a new population;
8. Determine whether the algorithm meets the termination conditions (minimizing the prediction error of CaO activity). If satisfied, proceed to (9); otherwise, proceed to (6);
9. Obtain the optimal hyperparameters (learning_rate, depth, n_estimators, l2_leaf_reg, subsample, bagging_temperature, colsample_bylevel);
10. Establish the model using the optimal hyperparameter combination;
11. Evaluate the IWOA-CatBoost model using the testing set;
12. Output the optimal hyperparameter combination (learning_rate, Depth, n_estimators, l2_leaf_reg, subsample, bagging_temperature, colsample_bylevel) and the model evaluation metrics (R^2^, RMSE, MAE).

### Improved whale optimization algorithm

Whale optimization algorithm (WOA) is an intelligent optimization algorithm proposed by Mirjalili et al.^28^, which has the characteristics of simple algorithm principle, few parameter setting and strong global search ability. The whale optimization process is divided into three main stages: encircling prey, bubble-net attacking method, and search for prey. The synergy of these three stages makes the whale optimization algorithm an effective tool for obtaining optimal solutions in different scenarios^29^.

1. Encircling prey.

The search range of the WOA algorithm is the entire solution space. Since the location of the optimal solution is unknown, a candidate solution is assumed to be the target prey. Once the prey is determined, the other whales update their positions to the target prey. This behavioral model is shown in Eq. (1).

1 $$\left\{ {\begin{array}{*{20}c} {X(t + 1) = X^{ * } (t) - AD} \\ {D = \left| {CX^{ * } (t) - X(t)} \right|} \\ \end{array} } \right.$$

where *t* represents the iteration number; X^*^(*t*) represents the position of the current optimal solution; *X*(*t*) represents the position of the whale; *X*(*t* + 1) represents the position of the whale at the next moment; *D* represents the distance between the position of the whale and the current optimal solution. *A* and *C* represent the parameters for updating the position of the whales, as shown in Eq. (2).

2 $$\left\{ {\begin{array}{*{20}c} {A = a(2r_{1} - 1)} \\ {C = 2r_{2} } \\ {a = 2(1 - t/t_{\max } )} \\ \end{array} } \right.$$

where *r*_1_ and *r*_2_ represent the random number in the range of [0, 1]; *t*_max_ represents the maximum number of iterations; *a* represents the convergence factor that linearly decreases from 2 to 0 as *t* increases.

(2) Bubble-net attacking method.

During the predation phase, the constriction encirclement and spiral ascent are performed simultaneously, bringing the prey close to the sea surface for hunting. This model of hunting behavior is shown in Eq. (3).

3 $$X(t + 1) = D \cdot e^{bl} \cdot \cos (2\pi l) + X^{ * } (t)$$

where, *b* is a constant for defining the shape of the logarithmic spiral; *l* is a random number in [− 1, 1].

(3) Search for prey.

The decision to perform either a global search (when |A|≥ 1) or a local search (when |A|< 1) is based on the value of |A|. When performing a global search, an individual whale is randomly selected to ensure the balance between local optimization and global search. The model is shown in Eq. (4).

4 $$\left\{ {\begin{array}{*{20}c} {X(t + 1) = X_{{{\text{rand}}}} (t) - AD_{1} } \\ {D_{1} = \left| {C \cdot X_{{{\text{rand}}}} (t) - X(t)} \right|} \\ \end{array} } \right.$$

where *X*_rand_ represents the position of a randomly selected whale individual within the population.

The above introduction is the standard WOA, where the balance between local optimization and global search significantly influences optimization accuracy and convergence speed. This balance is controlled by the parameter *A*. The primary factor influencing *A* is the convergence factor *a*, which linearly decreases from 2 to 0 as *t* increases in the standard WOA. This approach may lead to inadequate exploration of feasible solutions in the early stage and slow convergence in the later stage^30^. For this problem, a piecewise nonlinear convergence factor was proposed to improve both the exploration capability in the early stage and the convergence speed in the later stage, as shown in Eq. (5). Based on this, the IWOA algorithm is used to ensure a global search within the feasible solution by maintaining a large convergence factor with a slow reduction rate in the early iterations. In the later stages, the convergence factor is small, and its reduction rate is rapid to enhance the speed of local optimization.

5 $$\left\{ {\begin{array}{*{20}c} {a = 1 + \cos \left[ {\left( {\frac{{2t + t_{\max } }}{{2 \cdot t_{\max } }}} \right) \cdot \pi - \frac{\pi }{2}} \right],} & {t \le \frac{{t_{\max } }}{2}} \\ {a = 1 + \cos \left[ {\left( {\frac{{2t - t_{\max } }}{{2 \cdot t_{\max } }}} \right) \cdot \pi + \frac{\pi }{2}} \right],} & {t > \frac{{t_{\max } }}{2}} \\ \end{array} } \right.$$

### Categorical boosting (CatBoost) algorithm

CatBoost is an algorithm developed by the Russian company Yandex, based on oblivious trees as its base learners. In the boosting algorithms, CatBoost demonstrates higher computational accuracy and shorter training times compared to XGBoost. Furthermore, CatBoost effectively addresses the overfitting issue present in LightGBM through its ordered boosting method^31^. Therefore, a prediction model of a(CaO) was established based on CatBoost.

CatBoost has the following characteristics^32^: (1) It utilizes the Ordered Target-based Statistics method for feature label classification, employing a core principle of ranking to randomly permute the data through various methods, thereby generating different permutation sequences. Subsequently, for each permutation sequence, the average target value of samples belonging to the same category is calculated by estimating each sample. When handling the categorical features of each sample, the average target value of the previous categorical labels of that sample is utilized and presented in the form of numerical variables. This approach enhances the modeling capability of categorical features. (2)To solve the problem of gradient estimation bias, the step size of the gradient is improved in the first stage by utilizing unbiased estimates and employing the ordered boosting method for gradient calculation and estimation; In the second stage, the traditional gradient boosting decision tree algorithm is used for optimization. This method can effectively reduce the bias caused by gradient estimation, thereby solving the problem of prediction shift and improving the accuracy and generalization ability of the model.

### Model evaluation

Coefficient of determination (R^2^), root mean square error (RMSE), and mean absolute error (MAE) are adopted as the performance evaluation criteria for different models^33^. Table 2 shows the performance evaluation criteria.

**Table 2:** Performance evaluation criteria.

No.	Performance metric	Formulation
1	R^2^	${\text{R}}^{2} = \frac{{\sum\nolimits_{i = 1}^{{N_{p} }} {(y_{i}^{\exp } - \overline{{y_{m} }} )^{2} - \sum\nolimits_{i = 1}^{{N_{p} }} {(y_{i}^{{{\text{cal}}}} - y_{i}^{\exp } )^{2} } } }}{{\sum\nolimits_{i = 1}^{{N_{p} }} {(y_{i}^{\exp } - \overline{{y_{m} }} )^{2} } }}$
2	MAE	${\text{MAE}} = \sum\nolimits_{i = 1}^{{N_{P} }} {\left| {y_{i}^{{{\text{cal}}}} - y_{i}^{\exp } } \right|} /N_{P}$
3	RMSE	${\text{RMSE}} = \sqrt {\sum\nolimits_{i = 1}^{{N_{P} }} {(y_{i}^{{{\text{cal}}}} - y_{i}^{\exp } )^{2} } /N_{P} }$

*N*_*p*_ is the number of data, *y *^*exp*^ is the experimental value, *y *^*cal*^ is the calculated value, and $\overline{{y_{m} }}$ is the average value.

## Results and discussion

### Hyperparameter optimization of CatBoost

The Improved Whale Optimization Algorithm (IWOA) was used to explore and optimize specific hyperparameters of CatBoost, with the corresponding ranges and optimal values shown in Table 3. Learning rate (learning_rate): The learning rate is used to control the convergence speed of the algorithm. A smaller learning rate can make the model more stable but may require more training iterations to reach optimal performance. Depth of tree (depth): The depth of a tree refers to the maximum depth of each tree. Increasing the depth of the tree can improve the complexity of the model, thereby enhancing its performance. However, if the depth of the tree is too large, it may lead to overfitting. Number of tree (n_estimators): The number of trees refers to the number of trees in the model. Increasing the number of trees can enhance the model’s complexity, thereby improving its performance. However, if the number of trees is too large, it may lead to overfitting. L2 regularization coefficient (l2_leaf_reg): The L2 regularization coefficient is used to control the degree of regularization in the model. In general, smaller values of l2_leaf_reg tend to make the model more prone to overfitting, while larger values of l2_leaf_reg tend to make the model more prone to underfitting. Subsample ratio (subsample): The main purpose of the subsample parameter is to reduce overfitting by randomly selecting a portion of the data to simulate the diversity of the training set. Bagging temperature (bagging_temperature): The Bagging parameter is used to control the proportion of samples in each iteration step. A smaller Bagging parameter can reduce the variance of the model, thereby improving its stability. Feature sampling ratio (colsample_bylevel): The feature sampling ratio refers to the proportion of features considered for splitting at each node. A smaller feature sampling ratio can reduce the model’s variance, thereby improving the model’s stability^34^. Other hyperparameters were set to their default values as provided by Python’s CatBoost library. This approach aimed to improve model performance by focusing optimization efforts on key parameters, while allowing default settings for other parameters.

**Table 3:** Best parameters for CatBoost within the parameter boundaries.

Parameters name	Parameters boundaries	Best parameters
learning_rate	(0.001, 0.5)	0.3276
depth	(3, 10)	8
n_estimators	(100, 1000)	684
l2_leaf_reg	(1, 10)	8.6729
subsample	(0.1, 1)	0.6301
bagging_temperature	(0, 1)	0.5256
colsample_bylevel	(0.1, 1)	0.8468

### Comparison of IWOA-CatBoost model with other models and methods

Different performance evaluation metrics were used to compare the MLR, MLP, and KNN models with the optimal IWOA-CatBoost model, as shown in Table 4.

**Table 4:** Performance evaluation of different models and methods.

Model /method	R^2^	RMSE	MAE
FactSage	0.5396	0.0130	0.0083
IMCT	0.4891	0.0095	0.0058
GA-BP	0.7575	0.0074	0.0050
IWOA-CatBoost	0.9200	0.0042	0.0030

Table 4 show the performance of the various models and methods. In Table 4, the R^2^ value of the IWOA-CatBoost model were better than those of the FactSage, IMCT, and GA-BP model. The IWOA-CatBoost model achieved the highest R^2^ value of 0.9200, lowest RMSE of 0.0042, and lowest MAE of 0.0030 in predicting the *a*(CaO). Meanwhile, the R^2^ value of the IWOA-CatBoost model of the *a*(CaO) was 0.3804 higher than those of the FactSage, 0.4309 higher than those of the IMCT, and 0.1625 higher than those of the GA-BP model, respectively. The RMSE and MAE values of the IWOA-CatBoost model of the *a*(CaO) were 0.0088 and 0.0053 lower than those of the FactSage, 0.0053 and 0.0028 lower than those of the IMCT, and 0.0032 and 0.0020 lower than those of the GA-BP model, respectively. The possible analysis of the above results is as follows: (1) FactSage is a thermodynamic calculation software widely used in metallurgy for slag system analysis. It utilizes relevant databases to calculate the thermodynamic properties of slag^35^. However, it may not capture the complex non-linear interactions between slag components as effectively as data-driven models like CatBoost. Moreover, Factsage’s predictive accuracy is highly dependent on the quality and extent of the thermodynamic databases it uses^36^, which may limit its applicability and accuracy in predicting CaO activity compared to machine learning models that incorporate real-time data for modeling and prediction. (2) IMCT is a classical model used for predicting slag properties, based on a transfer function that models the relationship between slag composition and activity. While it provides reasonable estimates in many cases, it is limited by its linear relationships and least square method, which can lead to less accurate predictions in complex slag systems where interactions are more intricate. (3) GA-BP is a hybrid model combining genetic algorithms for optimization and a backpropagation neural network for prediction. While GA-BP performs well in capturing non-linear relationships, its performance heavily depends on the fine-tuning of the network’s hyperparameters. Additionally, the genetic algorithm optimization may lead to overfitting or convergence to local minima, which could affect the model’s predictive accuracy^37^. (4) The IWOA algorithm is used to ensure a global search within the feasible solution by maintaining a large convergence factor with a slow reduction rate in the early iterations. In the later stages, the convergence factor is small, and its reduction rate is rapid to enhance the speed of local optimization. CatBoost’s ability to handle feature interactions and non-linearities makes it more adaptable to complex slag systems^32^. IWOA optimizes the hyperparameters of CatBoost, which reduces the risk of overfitting compared to GA-BP and avoids the reliance on thermodynamic databases like Factsage.

**Fig. 4:**
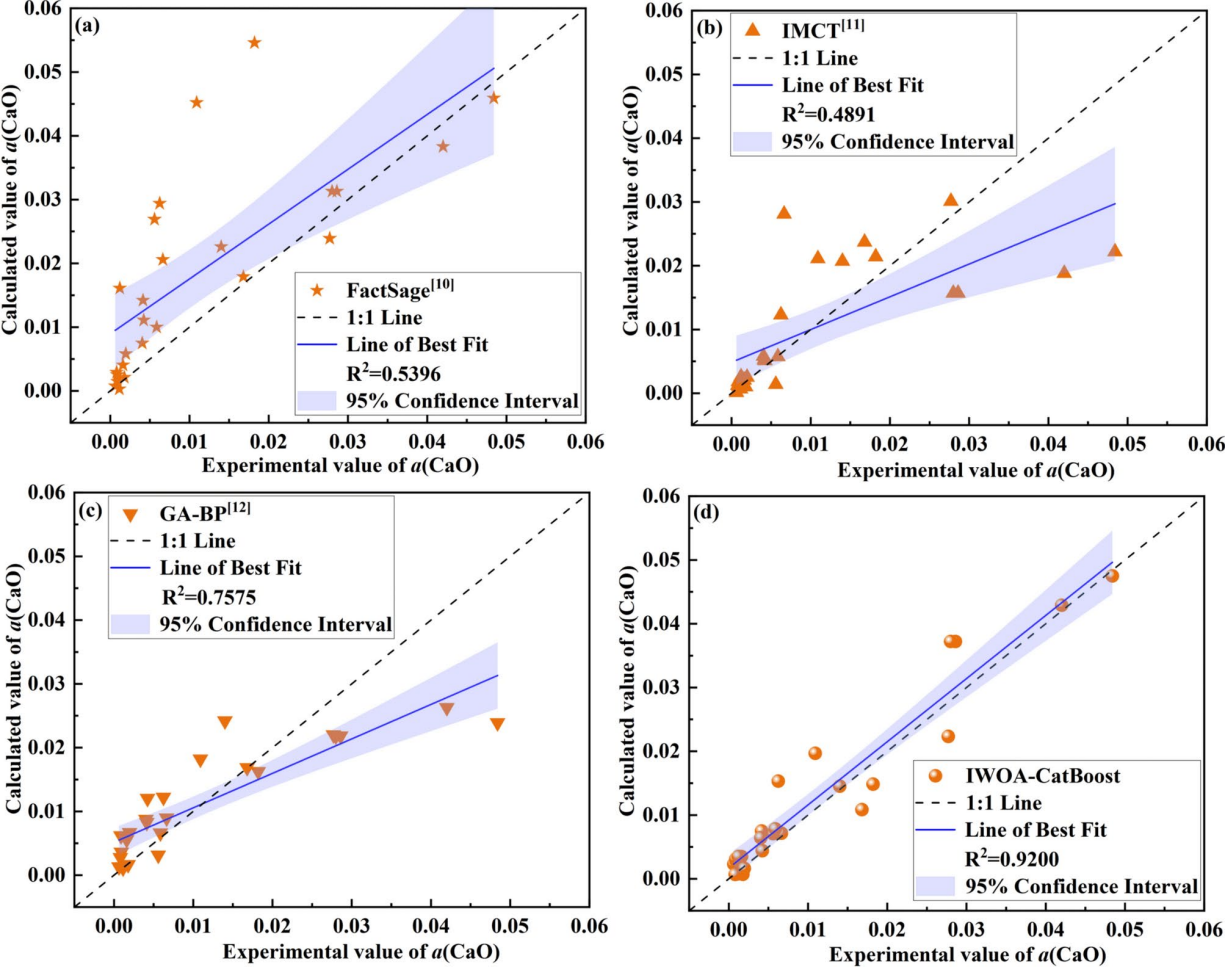
Comparison of the experimental and calculated *a*(CaO) on different models and methods using the same testing data set: (**a**) FactSage, (**b**) IMCT, (**c**) GA-BP, (**d**) IWOA-CatBoost.

In addition, the scatter plot, confidence interval, and absolute error plot were used to evaluate the performance of various models and methods. Figure 4 shows the comparison of the experimental and calculated *a*(CaO) on different models and methods using the same testing data set. The closer the scatter to the 45-degree diagonal line, the smaller the error between the calculated and experimental values. The coefficient of determination (R^2^) was used to evaluate the model’s goodness of fit, with the value closer to 1 indicating a stronger fitting ability. The confidence interval, shown as light blue shading and typically set at 95%, was used to reflect the uncertainty in the estimation results. A narrower confidence interval indicates greater stability in the model’s predictive performance^38^. In Fig. 4, the overall scatter plots of the IWOA-CatBoost prediction model of the *a*(CaO) was closer to the 45-degree diagonal dotted line than that of the FactSage, IMCT, and GA-BP model. Meanwhile, according to the R^2^ values, the IWOA-CatBoost model (R^2^ = 0.9200) demonstrates the strongest fitting ability, followed by the GA-BP model (R^2^ = 0.7575), whereas the FactSage and IMCT model display relatively weaker fitting performance. Additionally, as shown in Fig. 4, the IWOA-CatBoost model has the narrowest confidence interval, suggesting greater stability in its predictions. Based on various model evaluation metrics, the overall performance of the IWOA-CatBoost model is superior to that of the other models. Meanwhile, the regression line slope of the IWOA-CatBoost model is closer to 1 compared to other models, and the scatter distribution of predicted versus actual values is relatively concentrated, reflecting high prediction accuracy.

**Fig. 5:**
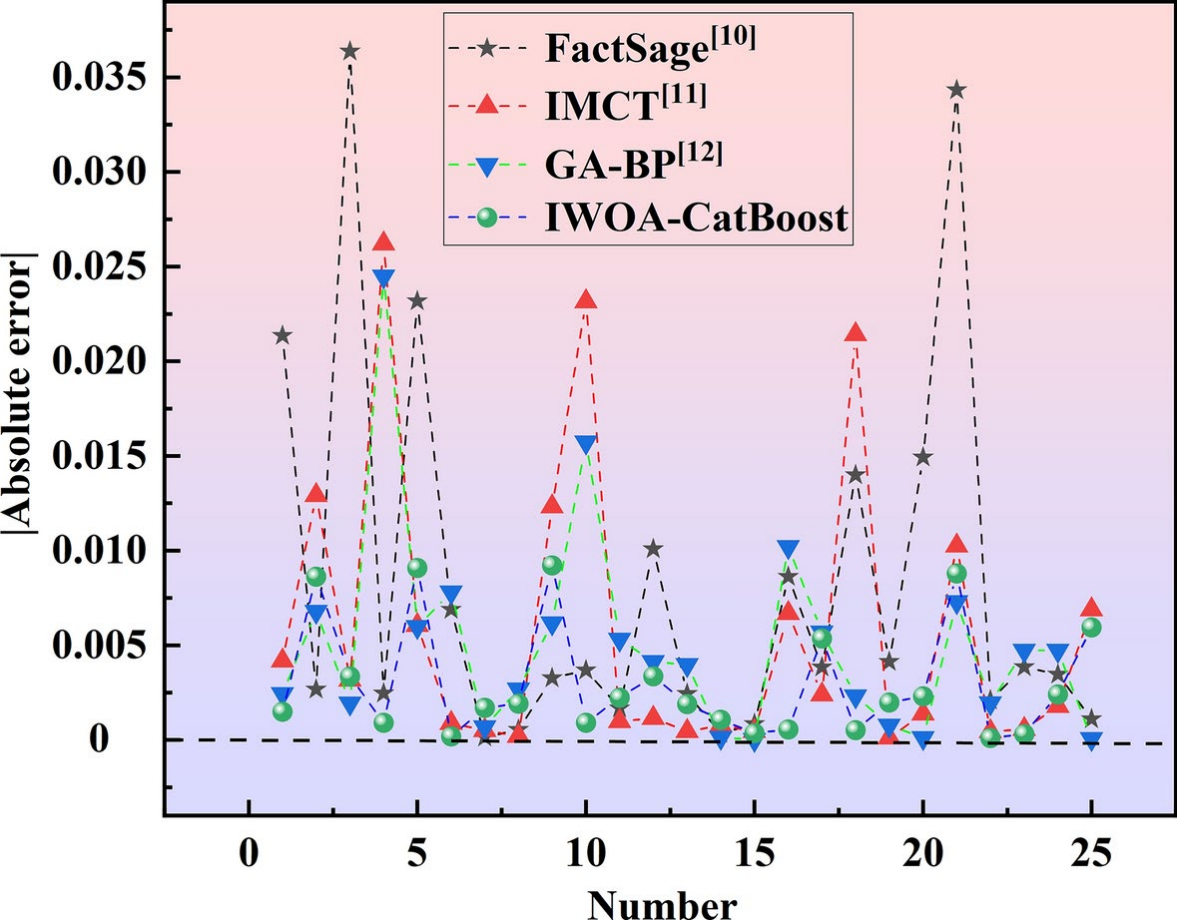
Comparison of the absolute values of the errors for different models and methods.

Figure 5 presents a comparison of the absolute values of the errors for different models and methods. In comparison with the FactSage, IMCT, and GA-BP, the absolute error of the a(CaO) value calculated by the IWOA-CatBoost model is overall closer to the zero reference line. Through an analysis of scatter plots, confidence intervals, and absolute error plots of different models, the IWOA-CatBoost model demonstrates advantages over the FactSage, IMCT, and GA-BP models in terms of confidence interval width, stability, fitting ability, and generalization performance. In the future, a dedicated activity database can be developed and data-sharing can be realized to further optimize the hyperparameters of the model established in this study. Meanwhile, the approach and algorithm used to develop the a(CaO) prediction model can also be applied to predicting the activity of other slag components or other metallurgical applications (e.g., predicting molten steel temperature, steel composition, and alloy yield), with stronger applicability, higher calculation accuracy and stronger generalization ability.

## Conclusions

Through the collection and analysis of experimental data of *a*(CaO), a feasible model was established to predict the *a*(CaO) using the IWOA-CatBoost. The following conclusions can be drawn.

1. Through correlation analysis, the effects of other variables on *a*(CaO) were listed in descending order of influence as follows: *w*(CaO), *w*(SiO_2_), temperature, *w*(MgO), and *w*(Al_2_O_3_). The optimal structures of the IWOA-CatBoost model had learning_rate of 0.3276, depth of 8, n_estimators of 684, l2_leaf_reg of 8.6729, subsample 0.6301, bagging_temperature of 0.5256, and colsample_bylevel of 0.8468.
2. The performance of the optimal IWOA-CatBoost model was evaluated and compared with that of existing models and methods. The IWOA-CatBoost model achieved the highest R^2^ value of 0.9200, lowest RMSE of 0.0042, and lowest MAE of 0.0030 in predicting the *a*(CaO), demonstrating superior stability, fitting accuracy, and generalization capability, thereby supporting its feasibility for calculating a(CaO). Meanwhile, the establishment method of *a*(CaO) prediction model can also be applied to the prediction of other slag components activity or other metallurgical applications.

## Data Availability

For data inquiries, please contact Zicheng Xin (sklxzc@163.com).
